# Increased Expression of Epidermal Growth Factor Receptor (EGF-R) in Patients with Different Forms of Lung Fibrosis

**DOI:** 10.1155/2013/654354

**Published:** 2013-06-09

**Authors:** Argyris Tzouvelekis, Paschalis Ntolios, Andreas Karameris, George Vilaras, Panagiotis Boglou, Andreas Koulelidis, Kostas Archontogeorgis, Konstantinos Kaltsas, George Zacharis, Evangelia Sarikloglou, Paschalis Steiropoulos, Dimitrios Mikroulis, Anastasios Koutsopoulos, Marios Froudarakis, Demosthenes Bouros

**Affiliations:** ^1^Department of Pneumonology, University Hospital of Alexandroupolis, Medical school, Democritus University of Thrace, 68100 Alexandroupolis, Greece; ^2^Department of Pathology, Veterans Administration Hospital (NIMTS), 11521 Athens, Greece; ^3^Department of Cardiothoracic Surgery, University Hospital of Alexandroupolis, Medical School, Democritus University of Thrace, 68100 Alexandroupolis, Greece; ^4^Department of Pathology, University Hospital of Alexandroupolis, Medical school, Democritus University of Thrace, 68100 Alexandroupolis, Greece

## Abstract

*Introduction*. Emerging evidence supports the role of epidermal growth factor-receptor (EGFR) in fibrogenesis. The aim of our study was to investigate the expression profiles of EGFR in three forms of IIPs, including idiopathic pulmonary fibrosis (IPF), cryptogenic organizing pneumonia (COP), and nonspecific interstitial pneumonia (NSIP). *Patients and Methods*. Twenty newly diagnosed patients with IPF, 15 with COP, and 15 with NSIP (cellular, *n* = 4 and fibrotic, *n* = 11) were investigated. Fifteen paraffin blocks obtained from the normal part of lungs removed for benign lesions were used as controls. Immunohistochemistry was carried out using specific monoclonal antibody. Results were verified by qRT-PCR. *Results*. A significant EGFR upregulation, both in protein and mRNA level, was observed in IPF, COP, and fibrotic NSIP samples compared to controls. EGFR was primarily localized in the hyperplastic alveolar epithelium surrounding areas of fibrosis in IPF, COP, and fibrotic NSIP samples, as assessed by double immunohistochemistry analysis with surfactant protein-A. EGFR mRNA levels were positively associated with indicators of lung fibrosis (type 1 collagen mRNA levels) and negatively correlated with functional prognostic parameters. *Conclusions*. We conclude that EGFR is upregulated in the hyperplastic alveolar epithelium in all three fibrotic forms of IIPs indicating a potential role during abnormal reepithelization.

## 1. Introduction

Idiopathic interstitial pneumonias is a heterogeneous group of diseases characterized by lung fibrosis. They share many common clinical and radiological features; however they differ in terms of underlying pathology and prognosis. Despite the increasing interest and effort during the last years from respiratory physicians and scientists, their etiology remains elusive and controversial. Additionally, most of them remain untreatable with a survival rate worse than that of many cancers [1].

The epidermal growth factor receptor (EGFR) is the cell surface receptor for members of the epidermal growth factor family (EGF family) of extracellular protein ligands. It is a member of the ErbB family of receptors, a subfamily of four closely related receptor tyrosine kinases: EGFR (ErbB-1), HER2/c-neu (ErbB-2), Her 3 (ErbB-3), and Her 4 (ErbB-4) [2, 3]. Upon activation by its growth factor ligands, EGFR undergoes a transition from an inactive monomeric to an active homodimer. EGFR dimerization leads to autophosphorylation and stimulation of tyrosine kinase pathways that ultimately initiate several downstream signaling pathways involved in cellular proliferation, differentiation, and apoptosis [2, 3]. EGFR is expressed in a variety of human tumors, including nonsmall cell lung cancer (NSCLC), breast, prostate, colorectal, bladder, and renal tumors [4]. Elevated EGFR levels are thought to promote tumor growth and treatment resistance. The latter notion is supported by studies showing increased EGFR expression in the metastatic forms of several tumors that exhibit substantial chemo- and radio resistance. In line with this premise, EGFR usefulness as potential biomarker of disease severity and treatment responsiveness has also been reported by several studies revealing that high EGFR levels were strongly associated with poor prognosis in patients suffering from several types of cancer including ovarian, breast, and pharyngeal [5–12]. On the other hand, the presence of a mutated EGFR receptor was found to be associated with a favorable response to certain types of EGFR inhibitors, namely, gefitinib and erlotinib in patients with lung adenocarcinoma [13–16].

While extensively investigated in patients with lung and other types of cancer, data arising by both human and experimental studies regarding its role and expression profile in different forms of pulmonary fibrosis is still conflicting and controversial. In particular, investigators showed that tyrosine kinase inhibitors that specifically inhibit EGFR or platelet-derived growth factor receptor (PDGFR) autophosphorylation could prevent collagen deposition (as assessed by increase in hydroxyproline accumulation) in a vanadium pentoxide-(V_2_O_5_-) induced fibrosis model [17]. Positive results were also reported by Hardie et al. in 2008, suggesting that EGFR inhibitors gefitinib and erlotinib prevent lung fibrosis in a TGF-*α*-induced fibrosis model [18]. Ishii et al. also reported that EGFR inhibitors including gefitinib can prevent bleomycin-induced fibrosis in mice [19]. These studies suggest a protective role for EGFR inhibitors in the course of lung fibrosis, indicating a potentially harmful contribution of the increased EGFR levels. On the other hand, another group of investigators supports an opposite role for EGFR inhibitors suggesting that inhibition of EGFR leads to augmentation and not amelioration of the bleomycin-induced fibrosis [20]. Interestingly, NSCLC patients' treatment with gefitinib has been associated with the development of pulmonary fibrosis [21–25]. A suggested mechanism is the inhibition of the EGFR-mediated alveolar regeneration [26, 27]. Most intriguingly, after therapeutic reversal of lung fibrosis, the reimplementation of gefitinib resulted in recurrence [28].

Taking into consideration the above and the urge for better understanding and treatment options for fibrotic lung disease, we sought to determine the expression profiles of EGFR in human disease and correlate our results with expression patterns of known markers of lung fibrosis (type 1 collagen) and functional parameters of disease severity including forced vital capacity (FVC) and diffusion lung capacity for carbon monoxide (DL_CO_). To authenticate the procedure, we utilized the pioneering technology of tissue microarrays that allowed us the simultaneous analysis of up to 50 human tissue samples from patients suffering from three different forms of IIPs: idiopathic pulmonary fibrosis (IPF), cryptogenic organizing pneumonia (COP), and nonspecific interstitial pneumonia (NSIP), cellular and fibrotic. 

## 2. Patients and Methods

### 2.1. Patients

A total of 50 patients diagnosed with one of the three IIPs were recruited for the study. Diagnosis was established using the ATS/ERS criteria 2011 for IPF and 2002 for the rest IIPs (specific radiological and histopathologal pattern coupled with exclusion of known causes of interstitial lung disease, mainly collagen vascular disease, sarcoidosis, and hypersensitivity pneumonitis based on history of exposure) [1, 29]. Paraffin-embedded surgical lung specimens (video-assisted thoracoscopic surgery: VATS) from two different fibrotic regions of each individual were sampled. Approval of the Ethics Committee of the Democritus University of Thrace, Greece, was obtained (reference number 1669/2010). All patients signed an informed consent where they agreed to the anonymous usage of their biological samples for research purposes. Part of the biopsy tissue was used to establish a diagnosis and the rest was formalin fixed and paraffin embedded to be used for tissue microarray construction. Twenty patients were diagnosed with IPF, 15 with COP and 15 with NSIP, fibrotic (*n* = 11) or cellular (*n* = 4) (Table 1). As seen in Table 1, based on functional data, all patients, enrolled in our study, were of mild to moderate disease severity. Fifteen control paraffin blocks obtained from the normal part of lungs removed for benign lesions (cysts, granulomas) were collected from the archives of the Department of Pathology of Veterans Hospital, Athens, Greece.

## 3. Methods

### 3.1. Tissue Microarrays

A total of 65 lung tissue samples including 50 samples from patients with three different forms of pulmonary fibrosis and 15 control tissues extracted from the normal part of the lung removed for benign lesions were used to construct two tissue microarrays based on an already published protocol. Details can be found in Supplementary Material available on line at http://dx.doi.org/10.1155/2013/654354.

### 3.2. Immunohistochemistry

Immunohistochemistry was performed by using specific monoclonal antibodies for EGFR (Abcam Ltd, ab2430) based on a standardized protocol. To further analyze the cellular localization of EGFR expression in type I and type II alveolar epithelial cells double immunohistochemistry analysis for EGFR and SP-A (SFTPA1-Abbiotec-6F10) was undertaken as described previously with slight modifications using En Vision double stain system protocol for paraffin-embedded tissue sections. Details can be found in an online data supplement.

### 3.3. Quantitative Real-Time Reverse Transcriptase-Polymerase Chain Reaction (qRT-PCR)

To quantify* Egfr* and *collagen type I* (COL1A2) expression we performed qRT-PCR by using the following primers: (1) forward primer *Egfr*-1753F, 5′-TGCGTCTCTTGCCGGAAT-3; reverse primer *Egfr*-1823R, 5′-GGCTCACCCTCCAGAAGGTT-3′; and TaqMan probe *Egfr*-1773Tc, 5′-ACGCATTCCCTGCCTCGGCTG-3′ (GenBank accession number AY588246) [30] and (2) COL1A2 primer pair: 5′ AGA GGA CCA CGT GGA GAA AG 3′ and 5′ GGC CTG TGG GAC CAT CTT 3′, according to a standardized protocol. Details can be found in an online data supplement.

### 3.4. Computerized Image Analysis

In order to evaluate the immunohistochemistry results not in a qualitative way but in a unbias accurate way, we performed CIA by using a semiautomated system (Matrox II Card Frame Grabber Camera Microwave Systems (640 × 480), Microscope Olympus BX-50). We calculated the levels of staining intensity (s.i) for each of the cases in epithelial cells and fibroblasts within a 256-level scale: 0 (black)–255 (white). Measurements of immunostained cytoplasms were performed in 5 optical fields per case and at several magnifications. A macro (BasicProPlus) was implemented. According to this, all stained nuclei (DAB stained dark or more light brown objects) per case in the corresponding optical fields were measured and the final number was filed in excel sheets. Values were converted to reverse percentages. Staining intensity values were then converted to reverse percentages (reverse staining intensity = (1− staining intensity/256) × 100). 

### 3.5. Statistical Analysis

Statistical analysis was carried out using SPSS 17.0 software. Results are expressed as mean ± SD or median (range), unless otherwise indicated. One-way ANOVA was used to compare reverse staining intensity values between patients and controls. In addition, statistical significance was further verified by performing independent samples *t*-test. Results were corrected using the Bonferroni correction. Spearman's correlation was used to find relationship between pulmonary function parameters and qRT-PCR expression levels of EGFR, as well as EGFR and COL1A2 mRNA levels in IPF patients. A *P* value of <0.05 was considered as statistically significant.

## 4. Results

### 4.1. Increased Expression of EGFR in IIPs Is Primarily Localized in the Hyperplastic Alveolar Epithelium

**Figure 1:**
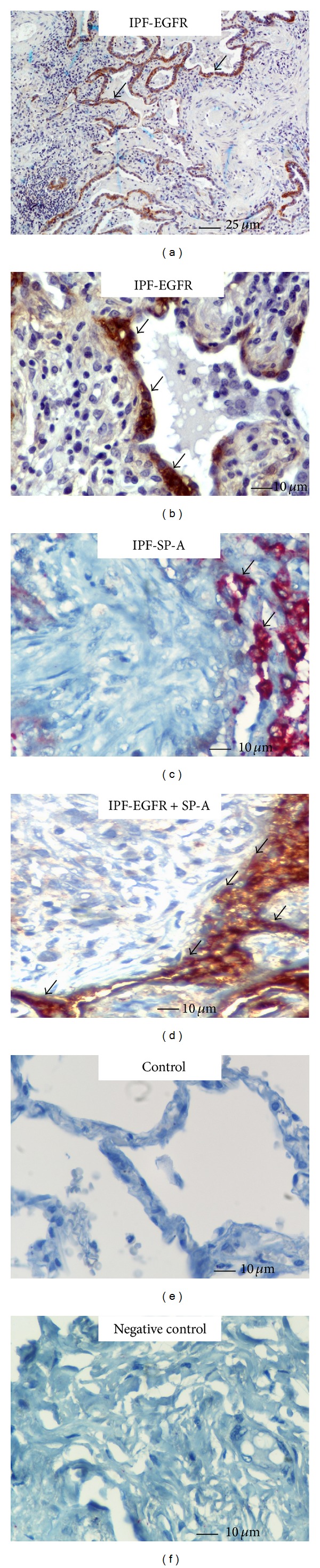
Representative tissue microarray section immunostained with monoclonal antibody EGFR demonstrating diffuse cytoplasmic reaction (dark brown) of strong intensity within hyperplastic alveolar epithelium (arrows) immediately adjacent to fibroblastic foci in patients with IPF (*n* = 20), evidence that was further verified by double immunohistochemistry analysis revealing strong colocalization (light brown with red) of EGFR and SP-A (arrows).

**Figure 2:**
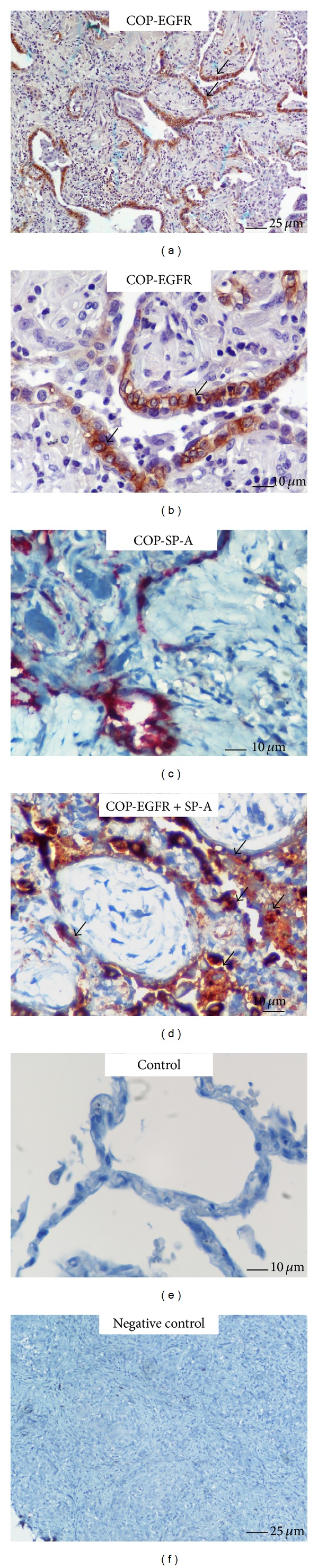
Representative tissue microarray section immunostained with monoclonal antibody EGFR demonstrating diffuse cytoplasmic reaction (dark brown) of strong intensity within hyperplastic alveolar epithelium (arrows) immediately adjacent to Masson bodies in patients with COP (*n* = 15), evidence that was further verified by double immunohistochemistry analysis revealing strong colocalization (light brown with red) of EGFR and SP-A (arrows).

**Figure 3:**
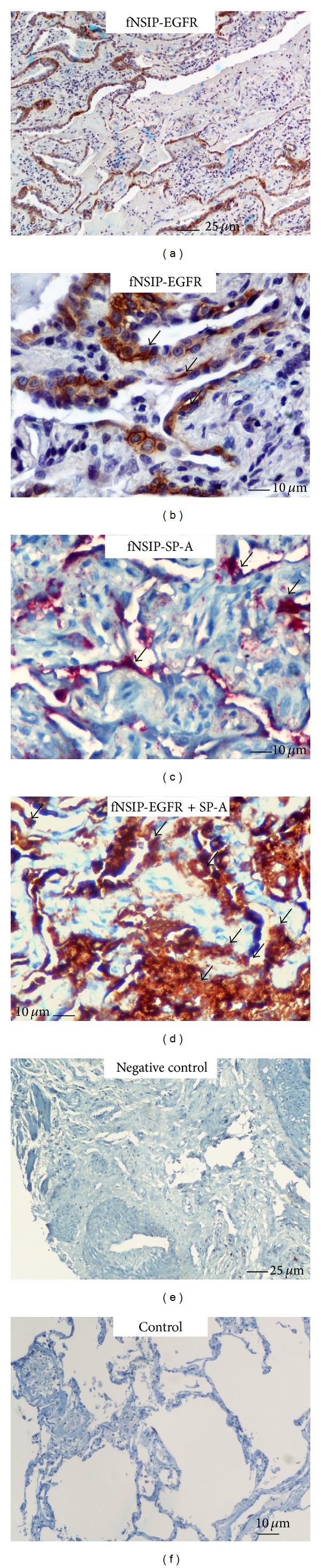
Representative tissue microarray section immunostained with monoclonal antibody EGFR demonstrating diffuse cytoplasmic reaction (dark brown) of strong intensity within hyperplastic alveolar epithelium (arrows) immediately adjacent to fibrotic areas in patients with fibrotic NSIP (*n* = 11), evidence that was further verified by double immunohistochemistry analysis revealing strong colocalization (light brown with red) of EGFR and SP-A (arrows).

**Figure 4:**
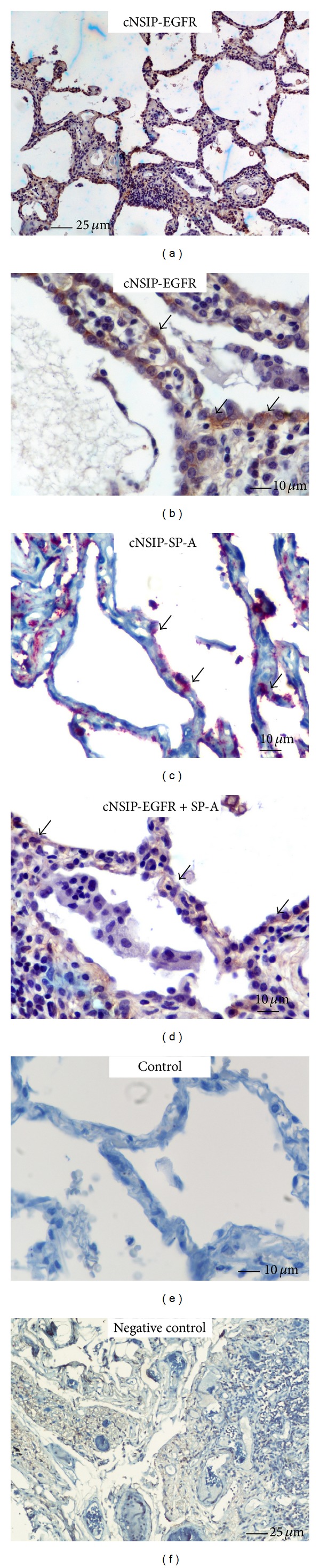
Representative tissue microarray section immunostained with monoclonal antibody EGFR demonstrating diffuse cytoplasmic reaction of weak intensity (light brown) within alveolar epithelium (arrows) immediately surrounding areas of inflammation in patients with cellular NSIP (*n* = 4), evidence that was further verified by double immunohistochemistry analysis revealing weak colocalization (light brown) of EGFR and SP-A (arrows).

**Figure 5:**
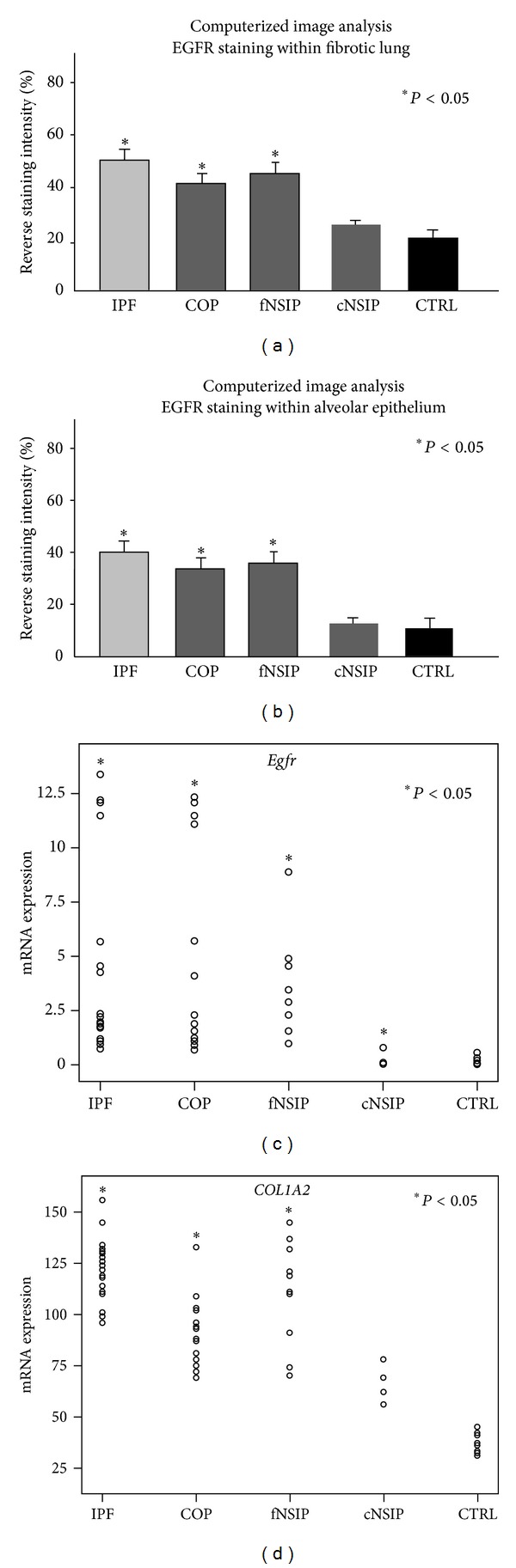
(a) Computerized image analysis verified results from immunohistochemistry analysis demonstrating a statistically significant increased expression of EGFR in patients with fibrotic forms of IIPs compared to the inflammatory component of cellular NSIP as well as control lung samples. *P < 0.05 (b) computerized image analysis also revealed a statistically significant increased expression of EGFR in the alveolar epithelium in IPF, COP, and fNSIP samples compared to cNSIP and normal alveolar epithelium. (c) Quantitative real-time PCR revealed a statistically significant upregulation of *Egfr* mRNA levels in patients with the fibrotic forms of IIPs including IPF, COP, and fibrotic NSIP compared to cellular NSIP and control lung samples. All values were normalized with the reference gene *B2m* and presented as relative expression to the control sample as described in materials and methods. *P < 0.05 (d) quantitative real-time PCR revealed a statistically significant upregulation of *COL1A2* mRNA levels in patients with the fibrotic forms of IIPs including IPF, COP, and fibrotic NSIP compared to cellular NSIP and control lung samples. All values were normalized with the reference gene *actin* and presented as relative expression to the control sample as described in materials and methods. *P < 0.05.

Immunohistochemistry staining and qRT-PCR were utilized to determine the EGFR expression profile, both in protein and mRNA level, in patients with three different forms of lung fibrosis. As seminally hypothesized, microscopic evaluation coupled with computerized image analysis of stained lung tissue samples revealed increased EGFR expression in the fibrotic forms of IIPs, comprising of IPF, COP, and fibrotic NSIP compared to the inflammatory component of cellular NSIP lung samples as well as control lung specimens (Figures 1–5(a)). To further analyze the cellular localization of EFGR within the fibrotic lung double immunohistochemistry analysis with SP-A was undertaken and strikingly revealed colocalization of EGFR with SP-A, indicating EGFR upregulation in alveolar type II epithelial cells mainly surrounding areas of fibrosis, including fibroblastic foci and Masson bodies (Figures 1-2). In addition, a strong colocalization of EGFR and SP-A within alveolar epithelium surrounding areas of inflammation and fibrosis in fNSIP samples was also noted (Figure 3). On the contrary, we observed weak colocalization staining intensity within alveolar epithelium surrounding areas of inflammation in cellular forms of NSIP (Figure 4). Macroscopic evaluation was further strengthened by computerized image analysis (Figure 5(b)). 

Moreover, immunostaining data was further supported by qRT-PCR which starkly demonstrated an upregulation of *Egfr *mRNA levels in 20 IPF (median values 4.41, ranges 0.93–13.4, *P* < 0.05), 15 COP (median values 4.65, ranges 0.68–12.34, *P* < 0.05) and 11 fibrotic NSIP (median values 4.35, ranges 0.97–8.9, *P* < 0.05), lung samples compared to 4 cellular NSIP (median values 0.22, ranges 0.01–0.09) and 10 control lung tissue specimens (median values 0.13, ranges 0.01–0.56) (Figure 5(c)). 

### 4.2. EGFR Expression Is Positively Correlated with Markers of Lung Fibrosis

**Figure 6:**
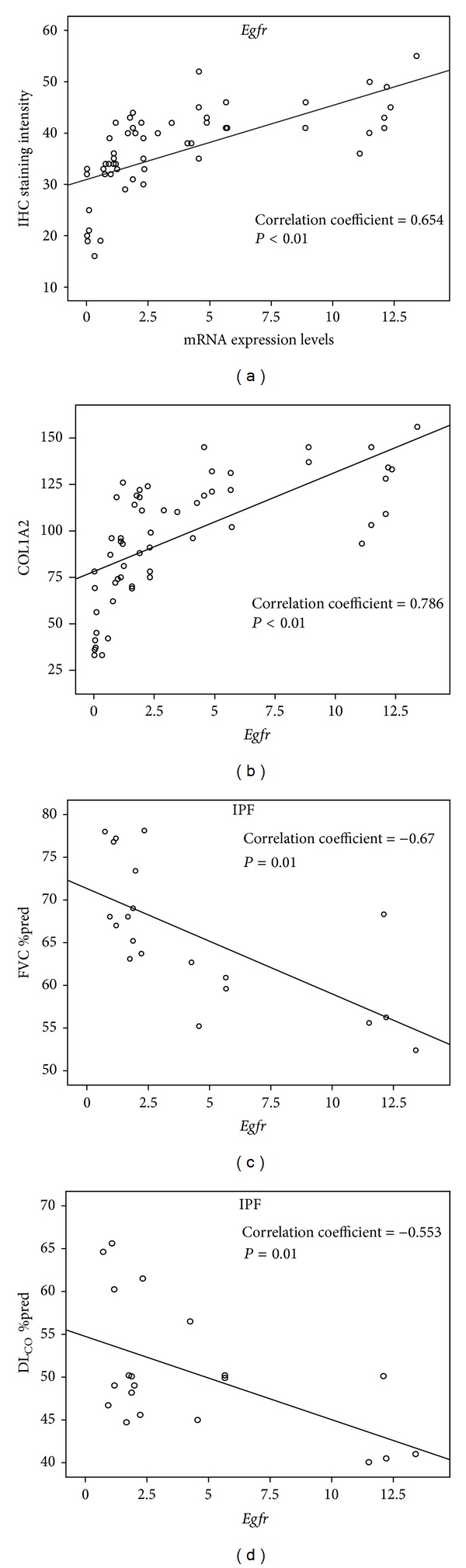
Spearman's correlation was performed and clearly demonstrated an almost linear positive association between *Egfr* mRNA levels and protein levels in patients with different IIPs (a), between *Egfr* and *COL1A2* mRNA in patients with different IIPs and finally a negative correlation between *Egfr* mRNA levels and functional parameters of disease severity and prognosis including forced vital capacity (FVC) (c) and diffusion lung capacity for carbon monoxide (DLCO) (d) in patients with IPF.

To further implicate EGFR upregulation in lung fibrosis we investigated the mRNA expression levels of type I collagen (COL1A2) in different types of IIPs. Importantly, qRT-PCR analysis demonstrated an expression pattern almost identical to EGFR, as estimated by increased mRNA levels in IPF, COP, and fNSIP lung specimens compared to cNSIP and control samples (Figure 5(d)). Moreover, statistical analysis revealed a significant positive association between EGFR (mRNA and protein) (Figure 6(a)) and COL1A2 mRNA expression levels in patients with IIPs (Figure 6(b)). 

### 4.3. EGFR Quantitative mRNA Expression Levels Were Negatively Correlated with Pulmonary Function Parameters in IPF Patients

To provide additional evidence that EGFR overexpression may contribute to the development and/or progression of pulmonary fibrosis we sought to correlate EGFR quantitative mRNA expression levels assessed by PCR analysis with functional parameters of disease severity, including FVC and DL_CO_. Statistical analysis revealed that an almost linear negative linkage was observed between EGFR mRNA expression levels and FVC, DL_CO_ (Figures 6(c) and 6(d)). On the other hand no correlations between EGFR mRNA expression levels and biomarkers of functional severity were noted in the other forms of lung fibrosis, namely, fNSIP, cNSIP, and COP (data not shown). 

## 5. Discussion

In this paper we present the expression profiles of EGFR in 3 distinct IIP entities for the first time in the literature. By using the pioneering technology of tissue microarrays coupled with immunohistochemistry and computerized image analysis and supported by qRT-PCR we managed to show the following: (1) increased expression of EGFR, both in protein and mRNA level, in lung tissue derived from the fibrotic forms of IIPs, comprising of IPF, COP, and fibrotic NSIP compared to the inflammatory component of cellular NSIP lung samples as well as control lung specimens, (2) EGFR abundant expression was colocalized with SP-A in the hyperplastic alveolar epithelium surrounding areas of fibrosis and inflammation in IPF, COP, and fNSIP samples, as estimated by dual immunostaining data, indicating a potential role for this receptor in the aberrant reepithelization that characterizes lung inflammation and fibrosis and (3) EGFR expression levels followed the same expression pattern with indicators of lung fibrosis, namely, COL1A2 while they were negatively correlated with functional parameters of disease severity and prognosis, namely, FVC and DL_CO_. The aforementioned evidence further implicates EGFR in lung fibrosis development and progression and highlights its potential usefulness as a reliable biomarker.

During the last 20 years, lung cancer and pulmonary fibrosis have been closely associated. Cigarette smoking, the major aetiologic factor for lung cancer, is thought to be a major risk factor for the development of IPF [31, 32]. Epithelial-mesenchymal transition (EMT) phenomenon and the apoptosis-resistant and proliferative properties of IPF fibroblasts are also found in cancer cells [33, 34]. Additionally, IPF patients develop lung cancer with increased probability compared with normal population [35]. Strengthening the above observations, EGFR, a well-established therapeutic target for lung cancer, is thought to play a role in the pathogenesis of pulmonary fibrosis. Its exact role is controversial, since animal experiments show both negative and positive effects from EGFR inhibition on fibrosis extent and severity [17–19]. However, the development of pulmonary fibrosis is a well-known adverse effect of the EGFR inhibitor gefitinib used in the treatment of lung adenocarcinoma [26, 27].

Our data show increased EGFR expression in the fibrotic forms of IIPs when compared with the inflammatory types of IIPs as well as normal controls. Intriguingly, EGFR expression was higher in the more severe types of fibrosis, indicating a harmful role of EGFR during the fibrotic process. In addition, EGFR was expressed mostly within the hyperplastic epithelium surrounding areas of fibrosis, as assessed by double immunohistochemistry analysis, revealing colocalization with SP-A. Our explanation for these results would be that EGFR is increased in an effort to repair fibrotic lesions through increased epithelial proliferation and differentiation, leading however to abnormal reepithelization. Whether this leads to amelioration or amplification of fibrosis may depend on the various mechanisms deregulated in lung fibrosis. It seems that the apoptotic function of EGFR activation is not enough to counter its proliferative, a notion strengthened by the lack of EGFR within the fibrotic lesions.

Finally, in an attempt to support our premise that EGFR upregulation may contribute to lung fibrosis and lead to more progressive disease stages, we have demonstrated that EGFR quantitative mRNA expression levels were positively correlated with expression levels of indicators of lung fibrosis such as COL1A2 while an almost linear negative association with markers of disease prognosis including pulmonary function parameters such as FVC and DL_CO_ was also shown. In addition, these linear correlations may indicate EGFR as a potential biomarker that could reliably predict clinical course and treatment response in IPF patients. However, future longitudinal studies in a large number of well-defined patients are sorely needed to support this provocative hypothesis.

Despite relative enthusiasm arising from our series of experiments, there is a number of limitations that should be addressed cautiously before rigid conclusions can be drawn. Firstly, it is worth noting that a major caveat of pathology studies is that the presented findings simply represent a snapshot of disease pathogenesis and by no means have they mirrored the entire pathogenetic cascade. It is therefore impossible to conclude a causal-effect relationship between EGFR upregulation and fibrogenesis. Secondly, our study was not designed to delineate mechanistic issues and elucidate the exact role of EGFR in different forms of lung fibrosis. Despite this, notions can be made based on the differences among the 3 IIPs and normal tissue, as well as the localization of EGFR in our tissue samples.

To sum up, this is the first report in the literature for EGFR levels in human biopsy samples comparing normal tissue with fibrotic lesions from IPF, NSIP, and COP. Our study builds upon current data on EGFR role in fibrosis and goes a step further, trying to suggest a possible role and results of EGFR activation. More studies are needed to delineate the role of EGFR in the pathogenetic cascade of abnormal wound repair leading to lung scarring and highlight potential benefits of EGFR as a therapeutic target for pulmonary fibrosis.

## Supplementary Material

Methods: Tissue Microarrays: Tissue samples were snap frozen and stored at 70° C. Specimens were fixed in cold-ethanol for 16 h and then embedded in paraffin. Tissue cylinders of 1.5 mm diameter were punched from selected areas of each “donor” block by utilizing a thin-wall stainless tube from a precision instrument (TMA-100, Chemicon, USA (29). Ultimately, we created two tissue microarray blocks comprising of 100 tissue elements each. Immunohistochemistry: The slides were deparaffinised and En Vision immunohistochemistry protocol (DAKI corp, Denmark) was carried through the use of an automated immunohistochemistry staining system (Bond-Biogenex, USA), as previously described (30-32). Briefly, this immunohistochemistry protocol is based on a water-soluble, dextran polymer system preventing the endogenous biotin reaction, which is responsible for the background in the stained slides. Quantitative Real-Time reverse transcriptase-polymerase chain reaction (qRT-PCR): RNA expression values (also referred to as relative mRNA expression) were calculated as ratios (differences between the Ct values) between Egfr and COL1A2 and B2M and b-actin, respectively, that served as housekeeping genes.

## Figures and Tables

**Table 1:** Baseline and functional characteristics of the study population.

Type of IIP	IPF	COP	fNSIP	cNSIP
Number of subjects	20	15	11	4
Male	18	9	8	2
Female	2	6	3	2
Mean age, years (range)	61 (44–80)	57 (48–72)	53 (48–61)	48 (40–58)
Smoking history				
Current smokers	0	0	0	0
Ex-smokers	20	12	7	3
FVC %pred	68 ± 14	75 ± 13	70 ± 9	80 ± 12
FEV_1_/FVC	88 ± 12	83 ± 5	86 ± 10	90 ± 11
TLC %pred	70 ± 9	74 ± 6	71 ± 8	81 ± 6
DL_CO_ %pred	52 ± 7	62 ± 5	56 ± 6	71 ± 7

Data are presented as median (range), no (total), or mean ± SD, unless stated otherwise.

cNSIP: cellular nonspecific interstitial pneumonia, COP: cryptogenic organizing pneumonia, DL_CO_: diffuse lung capacity for carbon monoxide, fNSIP: fibrotic nonspecific interstitial pneumonia, FVC: forced vital capacity, IPF: idiopathic pulmonary fibrosis, TLC: total lung capacity.

## Abbreviations

BALF: Bronchoalveolar lavage fluid
COP: Cryptogenic organizing pneumonia
EGFR: Epidermal growth factorreceptor
DL_CO_: Diffuse lung capacity for carbon monoxide
FEV_1_: Forced expiratory volume in one second
FVC: Forced vital capacity
IPF: Idiopathic pulmonary fibrosis
NSCLC: Nonsmall cell lung cancer
NSIP: Nonspecific interstitial pneumonia
PDGFR: Platelet-derived growth factor receptor
qRT-PCR: quantitative real-time polymerase chain reaction
TLC: Total lung capacity
VATS: Video-assisted thoracic surgery.
