# Supplementary material for: Increased Expression of Epidermal Growth Factor Receptor (EGF-R) in Patients with Different Forms of Lung Fibrosis
Source: Biomed Res Int. 2013 Jun 9;2013:654354. doi: 10.1155/2013/654354 (PMC3690645; doi:10.1155/2013/654354)
Supplement: Supplementary file 1 — Methods: Tissue Microarrays: Tissue samples were snap frozen and stored at 70° C. Specimens were fixed in cold-ethanol for 16 h and then embedded in paraffin. Tissue cylinders of 1.5 mm diameter were punched from selected areas of each “donor” block by utilizing a thin-wall stainless tube from a precision instrument (TMA-100, Chemicon, USA (29). Ultimately, we created two tissue microarray blocks comprising of 100 tissue elements each. Immunohistochemistry: The slides were deparaffinised and En Vision immunohistochemistry protocol (DAKI corp, Denmark) was carried through the use of an automated immunohistochemistry staining system (Bond-Biogenex, USA), as previously described (30-32). Briefly, this immunohistochemistry protocol is based on a water-soluble, dextran polymer system preventing the endogenous biotin reaction, which is responsible for the background in the stained slides. Quantitative Real-Time reverse transcriptase-polymerase chain reaction (qRT-PCR): RNA expression values (also referred to as relative mRNA expression) were calculated as ratios (differences between the Ct values) between Egfr and COL1A2 and B2M and b-actin, respectively, that served as housekeeping genes. [file 654354.f1.pdf]

Increased expression of epidermal growth factor-receptor (EGF-R) in patients with different forms of lung fibrosis

On line data supplement

Argyris Tzouvelekis[1], Paschalis Ntolios[1], Andreas Karameris[2], George Vilaras[2], Panagiotis Boglou[1], Andreas Koulelidis[1], Kostas Archontogeorgis[1], Konstantinos Kaltsas[1], George Zacharis[1], Paschalis Steiropoulos[1], Dimitrios Mikroulis[3], Anastasios Koutsopoulos[4], Marios Froudarakis[1] and Demosthenes Bouros[1]

[1]Department of Pneumonology, University Hospital of Alexandroupolis, Medical school, Democritus University of Thrace, Greece

[2]Department of Pathology, Veterans Administration Hospital (N.I.M.T.S), Athens, Greece

[3]Department of Cardiothoracic Surgery, University Hospital of Alexandroupolis, Medical School, Democritus University of Thrace, Greece

[4]Department of Pathology, University Hospital of Alexandroupolis, Medical school, Democritus University of Thrace, Greece

Correspondence:

Argyris Tzouvelekis, MD

Clinical Research Fellow, Medical School, Democritus University of Thrace.

Department of Pneumonology, University Hospital of Alexandroupolis

Alexandroupolis 68100

Tel & Fax: +30-25510-76106

Email: atzouvelekis@yahoo.gr

Methods

Tissue Microarrays

Tissue samples were snap frozen and stored at 70o C. Specimens were fixed in cold-ethanol for 16 h and then embedded in paraffin. Hematoxylin and eosin (H&E) – stained slides were used from each block to define the regions characterized by fibrotic lesions. Areas of interest were verified in H&E stained slides by using a conventional microscope (Olympus BX-50). Tissue cylinders of 1.5 mm diameter were punched from selected areas of each "donor" block by utilizing a thin-wall stainless tube from a precision instrument (TMA-100, Chemicon, USA) and were transferred by a solid stainless stylet into defined array coordinates in a 45 * 20 mm new recipient paraffin block (29). The tissue microarray blocks were constructed in three copies (each containing one sample from a different region of all lesions). One sample was taken from the centre and two samples from different peripheral areas. Ultimately, we created two tissue microarray blocks comprising of 100 tissue elements each. Each tissue element in the array was 2 mm in diameter and spacing between two adjacent elements as 0.1 mm. After the tissue microarray construction 3 μm sections for immunohistochemical analysis were cut from the "donor" blocks and were transferred to glass slides using an adhesive-coated tap sectioning system.

Immunohistochemistry

The slides were deparaffinised and En Vision immunohistochemistry protocol (DAKI corp, Denmark) was carried through the use of an automated immunohistochemistry staining system (Bond-Biogenex, USA), as previously described (30-32). Briefly, this immunohistochemistry protocol is based on a water-soluble, dextran polymer system preventing the endogenous biotin reaction, which is responsible for the background in the stained slides. More specifically, the sections were incubated with the primary antibody in "antibody diluent" (DAKO) and goat-anti-mouse EnVision-HRP-enzyme conjugate was performed for 3 min each. The "highly sensitive 3,3,' diaminobenzidine plus" (DAB+) and the "3- amino-9-ethylcarbazol plus" (AEC+) chromogens (both from DAKO) were used as substrates for the EnVision- HRP-

enzymes. Staining intensity was further enhanced by modifying the manufacturer's protocol in that all incubation steps (primary antibodies, EnVision, and substrate reactions) were performed on slides placed horizontally on a thermal plate at 37C. After each incubation the slides were dipped in TBS or, after the substrate reaction, in tap water at RT and waved at maximum speed for 10 sec. Excess liquid (buffer/water) was soaked up by a paper towel. Specimens of lung adenocarcinoma cases were used as positive controls for the marker. Specimens of human fibrotic lung were used as negative controls in each case in order to test for the specificity of the antibody involved.

Quantitative Real-Time reverse transcriptase-polymerase chain reaction (qRT-PCR)

RNA expression values (also referred to as relative mRNA expression) were calculated as ratios (differences between the Ct values) between *Egfr and COL1A2* and *B2M* and b-actin, respectively, that served as housekeeping genes and provided us with a normalization factor for the amount of RNA isolated from a specimen. qRT-PCR was performed using the Chromo 4 Real-Time Detection System and the Platinum® SYBR® Green qPCR SuperMix-UDG (Invitrogen), according to the manufacturer's instructions. The program used included: 2 min at 50°C, 5 min at 95°C, 43 cycles of denaturation-annealing- extension (30s at 95°C; 45s at 56°C; 30s at 72°C) and a final extension of 5 min at 72°C. Primers were chosen from exons separated by large introns (spanning exon-exon junctions), and the PCR quality and specificity was verified by melting curve analysis and gel electrophoresis.
